# Diurnal Blood Pressure Profiles and Hypertension-Mediated Organ Damage in Early Stages of Chronic Kidney Disease

**DOI:** 10.3390/life15121796

**Published:** 2025-11-24

**Authors:** Agnieszka Pluta, Paweł Stróżecki

**Affiliations:** 1 Division of Community Nursing, Faculty of Health Sciences, Ludwik Rydygier Collegium Medicum in Bydgoszcz of the Nicolaus Copernicus University in Toruń, Łukasiewicza 1, 85-821 Bydgoszcz, Poland; 2 Department of Nephrology, Hypertension and Internal Diseases, Faculty of Medicine, Ludwik Rydygier Collegium Medicum in Bydgoszcz of the Nicolaus Copernicus University in Toruń, Curie Skłodowskiej 9, 85-094 Bydgoszcz, Poland

**Keywords:** chronic kidney disease, blood pressure profile, hypertension-mediated organ damage, ambulatory blood pressure monitoring, cardiovascular risk

## Abstract

Introduction: Chronic kidney disease (CKD) is associated with a high risk of cardiovascular complications and mortality. This study aimed to assess the relationship between the diurnal blood pressure (BP) profile, progression of CKD, and hypertension-mediated organ damage (HMOD) in patients with CKD stages 1–3 during a 6-month observation period. Methods: Eighty-seven patients with CKD stages 1–3 underwent 24 h ambulatory blood pressure monitoring (ABPM), echocardiography, carotid intima-media thickness (IMT) assessment, and aortic pulse wave velocity (PWV) measurement at baseline and after 6 months. Serum creatinine and the estimated glomerular filtration rate (eGFR) were evaluated using the CKD-EPI formula. Results: Based on ABPM, patients were classified as dippers/extreme dippers (D/ED, 35.6%), non-dippers (ND, 47.2%), and reverse dippers (RD, 17.2%). At follow-up, the RD group showed a significant decline in the eGFR and a lower left ventricular ejection fraction compared to D/ED. IMT values were consistently higher in RD than in D/ED at baseline and follow-up. No significant differences in PWV were observed. Conclusions: An abnormal diurnal BP profile is common in patients with CKD stages 1–3. The “reverse dipper” profile is associated with faster CKD progression, more advanced vascular remodeling, and reduced left ventricular function. The results of our study support the role of ABPM as a useful tool in assessing risk in the early stages of CKD, providing additional prognostic information beyond office blood pressure measurements. Limitations: The relatively small sample size, short follow-up period, lack of detailed data on treatment modifications, and absence of orthostatic BP assessment may limit the interpretation and generalizability of the results.

## 1. Introduction

Chronic kidney disease (CKD) is a major global health problem, affecting nearly 10% of the adult population worldwide, and is strongly associated with increased cardiovascular morbidity and mortality [1,2]. Cardiovascular disease remains the leading cause of death among patients with CKD, even in its early stages, highlighting the need for early risk stratification and targeted interventions [3].

Hypertension is both a cause and a consequence of CKD and affects the vast majority of patients as kidney function declines [4,5]. The coexistence of hypertension accelerates the progression of renal impairment and contributes to the development of hypertension-mediated organ damage (HMOD) [6].

Hypertension-mediated organ damage (HMOD) encompasses structural and functional changes in the heart, vasculature, kidneys, and other organs resulting from chronic elevation of blood pressure. HMOD is a marker of subclinical cardiovascular disease and a strong predictor of adverse outcomes, particularly in patients with CKD [6]. Echocardiographic parameters, carotid intima-media thickness (IMT), and arterial stiffness assessed by pulse wave velocity (PWV) are commonly used surrogate markers of HMOD in both clinical practice and research [6,7,8]. Early identification of HMOD in CKD patients may provide additional prognostic information beyond conventional risk factors and could guide more personalized therapeutic strategies.

Ambulatory blood pressure monitoring (ABPM) allows for a more accurate assessment of blood pressure control compared with office measurements and is particularly valuable in CKD. ABPM not only provides prognostic information regarding cardiovascular outcomes but also identifies abnormal diurnal blood pressure (BP) patterns, which are common in CKD patients [9,10].

Abnormal diurnal BP profiles, including non-dipping and reverse-dipping patterns, have been associated with faster progression of kidney dysfunction and increased cardiovascular complications [11,12,13]. Recent data confirm that reduced or absent nocturnal BP decline is an independent predictor of CKD progression and cardiovascular events [14]. However, evidence remains limited regarding the impact of diurnal BP profile on early-stage CKD (stages 1–3) and its association with hypertension-mediated organ damage.

Therefore, the aim of this study was to evaluate the relationship between diurnal BP profiles, CKD progression, and HMOD in patients with CKD stages 1–3 during a 6-month follow-up period.

## 2. Material and Methods

The study was performed between the years 2012 and 2014. Approval was obtained from the Bioethics Committee at the Ludwik Rydygier Collegium Medicum in Bydgoszcz of the Nicolaus Copernicus University in Toruń.

The study included patients receiving treatment at the Nephrology Outpatient Clinic of University Hospital No. 1 in Bydgoszcz. Inclusion criteria for the study were: age above 18 years, CKD stages 1–3, and informed consent to participate in the study. Exclusion criteria included diabetes and ongoing immunosuppressive treatment.

A total of 130 patients were invited to participate in the study, of which 40 declined. Among the 90 participants, 30 were diagnosed with CKD stage 1, 33 with CKD stage 2, and 27 with CKD stage 3. Three individuals did not complete the study according to the protocol: 1 woman due to pregnancy and 2 patients due to failure to attend the follow-up examination after 6 months. Thus, for the purposes of this study, the results of 87 CKD patients were analyzed both at the beginning of the observation period and after 6 months.

In the study population, the causes of CKD were as follows: chronic glomerulonephritis confirmed by kidney biopsy (n = 16; 18.4%), hypertensive nephropathy (n = 3; 3.5%), polycystic kidney disease (n = 28; 32.2%), nephropathy in the course of gout (n = 5; 5.7%), kidney stone disease (n = 23; 26.4%), nephrectomy due to trauma (n = 1; 1.1%). In 11 cases (12.6%), the cause of the disease could not be determined.

All study participants underwent the following assessments at the beginning of observation and after 6 months: determination of serum creatinine concentration, estimation of eGFR using the CKD-EPI formula [15], and 24 h ambulatory blood pressure monitoring (ABPM) using the A&D Medical TM-2430 device. The cuff size was selected for each patient based on their arm circumference. The patients were instructed on how to operate the device. ABPM measurements were taken every 30 min. The duration of day and night was arbitrarily defined as lasting between 6:00 a.m. and 9:59 p.m. and between 10:00 p.m. and 5:59 a.m., respectively. Only readings with at least 80% valid measurements were used for analysis.

Based on the ABPM results, the average values of blood pressure were calculated as follows: systolic blood pressure (SBP) and diastolic blood pressure (DBP) over the entire day (SBP 24 h, DBP 24 h), during the daytime (SBP day, DBP day), and at night (SBP night, DBP night). The percentage decrease in systolic blood pressure was calculated using the following equation:

% SBP decrease = [(SBP day − SBP night)/SBP day] × 100%.

If the relative decrease in SBP at night was at least 10% and less than 20%, the patient was classified as a “dipper”. If the SBP decrease at night equaled or exceeded 20%, the patient was classified as an “extreme dipper”. In the case where the SBP decrease at night was less than 10%, the patient was classified as a “non-dipper”. If SBP at night increased compared to the daytime, the patient was classified as a “reverse dipper” [11].

All study participants underwent echocardiographic examinations measuring the end-diastolic thickness of the interventricular septum (IVSd), the left ventricle (LVIDd), and the posterior wall of the left ventricle (PWd). The measurements were performed in accordance with recommendations of the American Society of Echocardiography [16]. Left ventricular mass (LVM) was calculated using the formula proposed by Devereux et al.:

LVM = 0.8 × [1.04(IVSd + LVIDd + PWd)^3^ − LVIDd^3^] + 0.6 (g).

The LVM result was then applied together with body surface area (BSA) to calculate the left ventricular mass index (LVMI) as [17]:

LVMI = LVM/BSA.

Relative wall thickness (RWT) was also calculated as:

RWT = 2 × PWd/LVIDd.

Ejection fraction (EF) was calculated from the 2D apical four-chamber view using the area-length method.

The measurement of aortic pulse wave velocity (PWV) was performed between the carotid artery and the femoral artery using the Complior apparatus (Artech Medical, Pantin, France). The examinations were conducted on an empty stomach, in a quiet room, after a 10 min rest, in the supine position. One of the sensors of the apparatus was placed at the palpable pulse site on the right carotid artery, while the second sensor was placed at the palpable pulse site on the right femoral artery. The time (t) between the appearance of the pulse wave on the carotid artery and the femoral artery was measured automatically over 10 consecutive cycles and averaged. The distance of the pulse wave (d) was taken as the distance between the points where the sensors were applied on the carotid and femoral arteries, multiplied by a coefficient of 0.8, in accordance with current recommendations [18]. PWV was calculated using the equation PWV = d/t and expressed in [m/s]. Each patient underwent 2 measurements of PWV, one immediately after the other, and then the average was calculated.

All subjects underwent an ultrasound measurement of common carotid artery intima-media thickness (IMT). Patients were examined in the supine position after a 5 min rest. The thickness of the IMT complex was measured 10–30 mm below the carotid artery bifurcation at 3 points free from atherosclerotic plaques, on the right and left sides.

The arithmetic mean was calculated based on the obtained measurement results.

Each patient underwent a fasting blood draw for laboratory testing, which included the determination of serum creatinine concentration, total cholesterol, and triglycerides.

The duration of AH was determined on the basis of medical history and documentation analysis.

### Statistical Analysis

Statistical analysis was performed using Statistica v. 13 software (StatSoft, Tulsa, OK, USA). The normality of the distribution of variables was checked using the Shapiro–Wilk test. Variables with a normal distribution were presented as the mean ± standard deviation (SD) and compared using Student’s *t*-test. To compare more than 2 variables, the ANOVA test was employed. Post hoc analysis was conducted using Tukey’s test. Relationships between the analyzed parameters were assessed using the Pearson linear correlation coefficient. The level of statistical significance was set at *p* < 0.05.

## 3. Results

**Figure 1:**
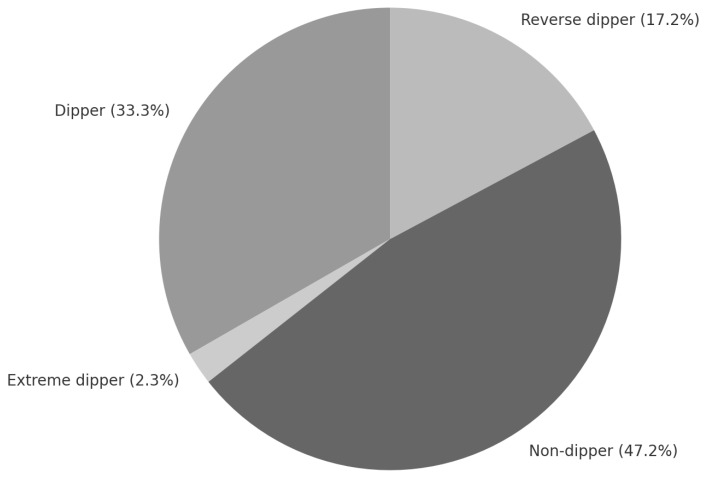
Diurnal blood pressure profiles in the analyzed group of stage 1–3 CKD patients.

The clinical characteristics of patients and study results are presented in Table 1. The study was conducted in a group of 87 patients, 54% of whom were male and 46% were female. Characteristics of blood pressure profile referred to as “dipper” (D) were observed in 33.3% of the patients, “extreme dipper” (ED) in 2.3% of the patients, “non-dipper” (ND) in 47.2% of the patients, and “reverse dipper” (RD) in 17.2% of the patients (Figure 1). Due to the low number of ED profile patients, D and ED groups were analyzed together.

**Figure 2:**
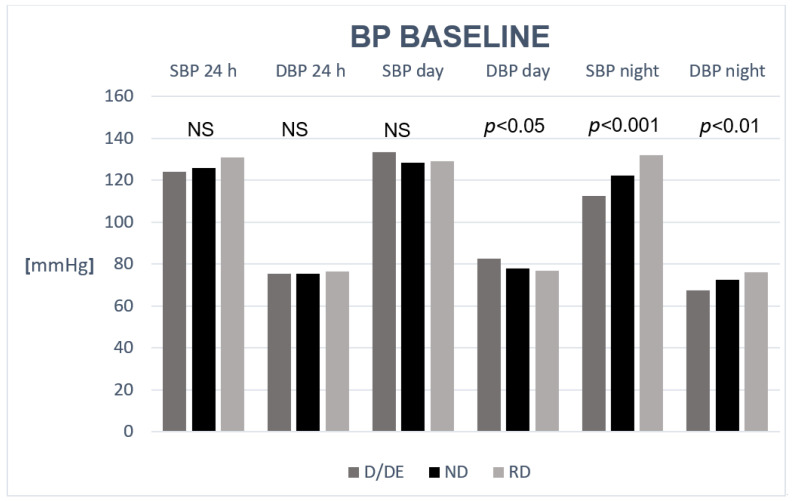
Comparison of blood pressure results in stage 1–3 CKD patients in the dipper + extreme dipper (D/ED), non-dipper (ND), and reverse dipper (RD) groups at baseline.

A comparison of blood pressure results in CKD patients in the D/ED, ND, and RD groups at the start of the study is presented in Figure 2.

**Figure 3:**
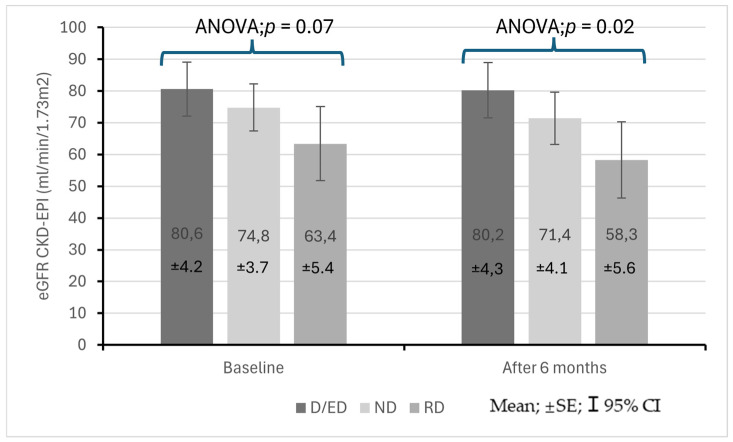
Changes in eGFR in stage 1–3 CKD patients at the start of observation and after 6 months with regard to baseline diurnal blood pressure profile.

**Figure 4:**
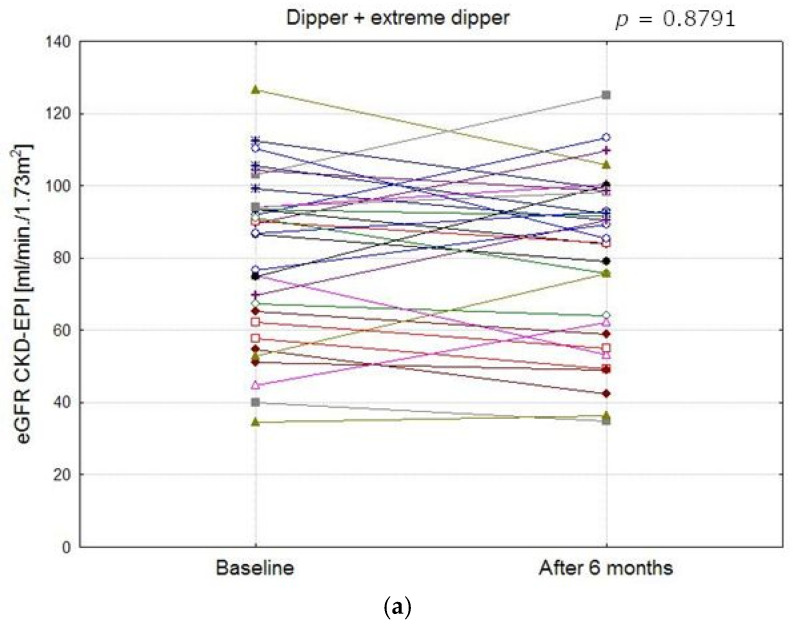

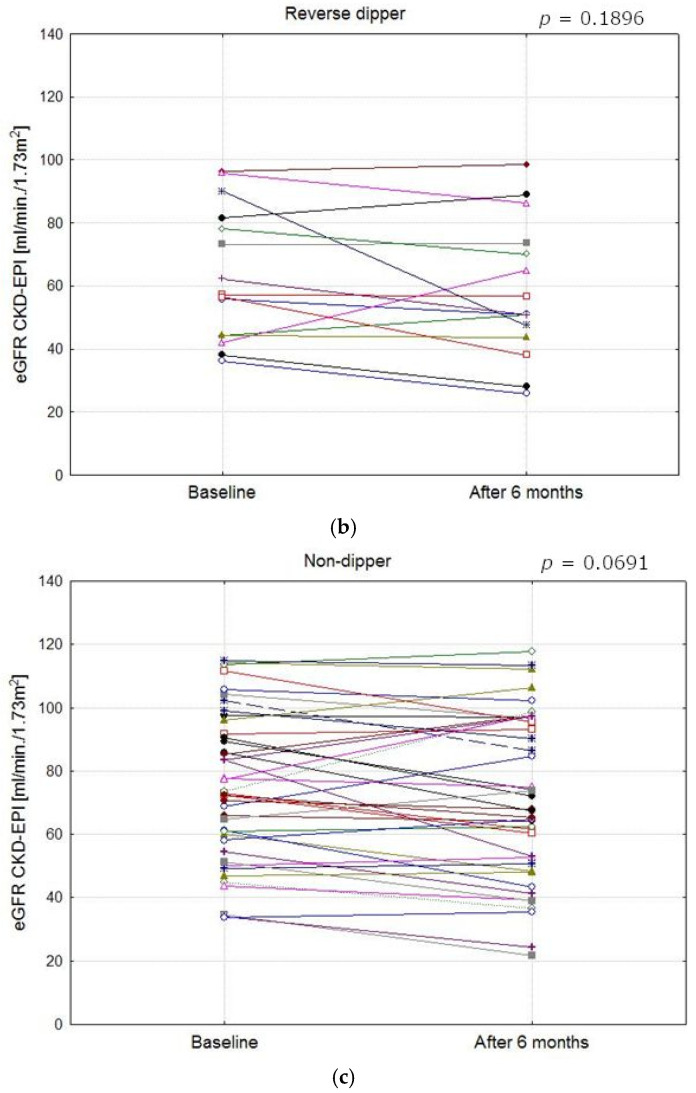
Comparison of eGFR CKD-EPI values in patients with different blood pressure profile groups at baseline and after 6 months (**a**–**c**). (**a**) eGFR CKD-EPI values in dipper and extreme dipper patients (The mean values at baseline and after 6 months did not differ significantly, *p* = 0.8791). (**b**) eGFR CKD-EPI values in reverse dipper patients (The mean values at baseline and after 6 months did not differ significantly, *p* = 0.1896). (**c**) eGFR CKD-EPI values in non- dipper patients (The mean values at baseline and after 6 months did not differ significantly, *p* = 0.0691).

Figure 3 presents changes in the eGFR in the study population at baseline and at follow-up. At the start of the observation period, the eGFR did not differ significantly between the groups (ANOVA *p* = 0.07), but after 6 months, the difference was statistically significant (ANOVA *p* = 0.02). At follow-up, differences in the eGFR between patients with the RD profile and those with the D/ED profile were found to be statistically significant (58.3 ± 21.81 vs. 80.2 ± 23.8 mL/min/1.73 m^2^; *p* = 0.02). Figure 4a–c show eGFR (CKD-EPI) values in patients from different blood pressure profile groups at baseline and after 6 months.

In the entire examined population, no statistically significant correlations were found between the difference in SBP day and night and eGFR values. However, in the RD group at baseline, we found a statistically significant negative correlation between the difference in SBP day and night and the eGFR (r = −0.58; *p* = 0.02).

At baseline, EF did not differ between study groups (ANOVA test; *p* = 0.69), but at follow-up, a significant difference in EF was found (ANOVA test; *p* = 0.03), with EF being significantly lower in the RD group compared to D/ED (58 ± 4 vs. 64 ± 6%; *p* < 0.05).

**Figure 5:**
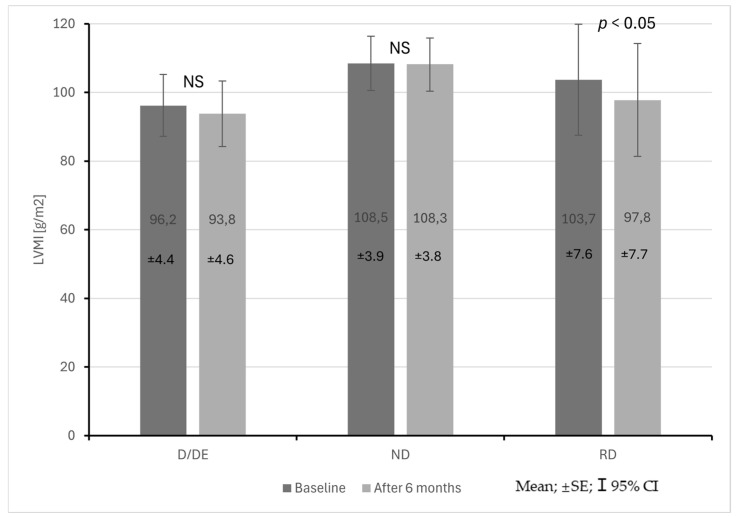
Changes in left ventricular mass index (LVMI) in CKD patients with dipper + extreme dipper (D/ED), non-dipper (ND) and reverse dipper (RD) blood pressure profiles during a 6-month observation period.

The RD patient group was also characterized by a statistically significant decrease in EF during the observation period (62 ± 8% vs. 58 ± 4%; *p* < 0.05). LVMI did not differ significantly between the groups both at baseline and at follow-up. In the RD patient group, a statistically significant decrease in LVMI was observed during the observation period (103.7 ± 29.3 vs. 97.8 ± 29.7 g/m^2^; *p* < 0.05) (Figure 5).

**Figure 6:**
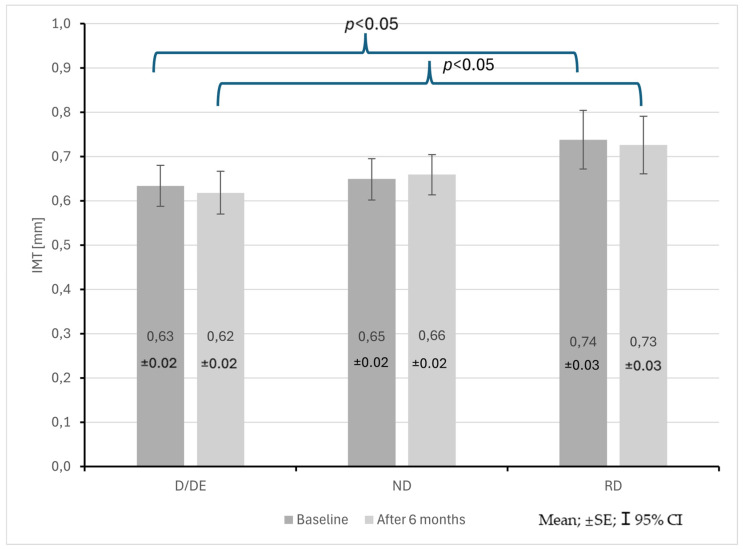
Changes in common carotid artery intima–media thickness (IMT) in CKD patients with dipper + extreme dipper (D/ED), non-dipper (ND) and reverse dipper (RD) blood pressure profiles during a 6-month observation period.

The group of patients with the RD profile also showed an increase in IMT compared to patients with the D/ED profile both at the start and after 6 months of observation (0.74 ± 0.12 mm vs. 0.63 ± 0.13 mm; *p* < 0.05, 0.73 ± 0.12 mm vs. 0.62 ± 0.13 mm; *p* < 0.05) (Figure 6). No statistically significant differences were found in PWV.

The results of multiple regression analysis for parameters of subclinical damage at baseline and after 6 months are presented in Table 2.

## 4. Discussion

In this study, abnormal diurnal blood pressure (BP) profiles were highly prevalent among patients with CKD stages 1–3, with the non-dipper pattern being the most frequent, followed by the reverse-dipper profile. These findings are consistent with earlier reports showing that abnormal circadian BP patterns are common in CKD patients, even at the early stages of the disease [10,12,13,19]. Importantly, the reverse-dipper profile was associated with a more pronounced decline in renal function and adverse cardiovascular surrogate markers during the 6-month follow-up.

The prognostic significance of diurnal BP profiles in CKD has been consistently reported. Both nondipping and reverse-dipping patterns are associated with faster progression of kidney dysfunction and increased cardiovascular risk [5,10,11,12,13,14]. In our cohort, patients with the reverse-dipper profile demonstrated a significant decline in eGFR compared with dipper/extreme-dipper patients. This observation aligns with prospective data indicating that nocturnal BP alterations independently predict renal function deterioration in CKD [5,11,19,20]. These findings further emphasize the clinical value of ambulatory blood pressure monitoring (ABPM), which consistently provides superior prognostic information compared with office measurements [9,10,19,20].

With regard to cardiac remodeling, patients with the reverse-dipper profile exhibited lower ejection fraction after 6 months, suggesting early impairment of left ventricular systolic function. This observation is supported by previous reports linking abnormal BP rhythms with increased cardiac workload and adverse remodeling [6,12]. Furthermore, carotid intima-media thickness (IMT) was higher in reverse-dippers both at baseline and follow-up. IMT is an established early marker of atherosclerosis and a predictor of cardiovascular outcomes in CKD [7,8]. Recent state-of-the-art reviews have underscored its strong predictive value for cardiovascular morbidity and mortality in this population [21]. Thus, our results suggest that abnormal BP profiles, particularly the reverse-dipper pattern, are associated with early hypertension-mediated organ damage (HMOD) involving both cardiac and vascular structures.

Interestingly, no significant differences in pulse wave velocity (PWV) were observed between groups. PWV is a recognized non-invasive marker of arterial stiffness and has been included in the 2023 ESH guidelines as a diagnostic criterion for subclinical organ damage when values exceed 10 m/s [6,18]. Although our results did not show group differences, this may be due to the relatively small sample size, short observation period, and lack of control for confounders such as age, mean arterial pressure, and metabolic abnormalities. Larger studies and contemporary reviews have consistently demonstrated that increased PWV is an independent predictor of cardiovascular complications and mortality in CKD patients [22,23].

Age is another important factor that requires consideration. In our study, reverse-dippers were significantly older than dipper/extreme-dipper patients, which may partly explain the differences in renal function, EF, and IMT. Previous studies have shown that advanced age is strongly associated with abnormal diurnal BP profiles and HMOD [12,13]. Moreover, orthostatic hypotension (OH), which is more common in elderly hypertensive patients, has been suggested as a contributor to the reverse-dipper profile. OH itself has been shown to predict cardiovascular events and may represent a confounding factor in interpreting our findings [24]. Unfortunately, OH was not assessed in the present study.

Several limitations should be acknowledged. First, the relatively small sample size and short follow-up period (6 months) limit the generalizability of our findings. Second, although all patients received pharmacological treatment according to standard clinical practice, changes in therapy during follow-up were not systematically recorded or controlled, which may have influenced BP control and HMOD parameters. Third, important potential confounders such as orthostatic hypotension, metabolic disturbances, and detailed comorbidity profiles were not evaluated. Finally, our findings are based on surrogate markers of cardiovascular risk rather than hard clinical endpoints.

In conclusion, ABPM enables the identification of abnormal diurnal BP profiles with important prognostic implications in CKD stages 1–3. Patients with the reverse-dipper profile demonstrated faster decline in kidney function and more advanced vascular remodeling, which may be partly age-related. These findings support the role of ABPM in the early risk stratification of CKD patients and highlight the need for future prospective studies to determine whether targeted therapeutic interventions in patients with abnormal BP profiles can improve renal and cardiovascular outcomes.

## 5. Conclusions

Abnormal diurnal blood pressure profiles are highly prevalent among patients with CKD stages 1–3. The reverse-dipper profile was associated with a faster decline in kidney function, higher carotid intima-media thickness, and lower left ventricular ejection fraction, suggesting early hypertension-mediated organ damage. The results of our study support the role of ambulatory blood pressure monitoring (ABPM) as a useful tool in assessing risk in the early stages of CKD, providing additional prognostic information beyond office blood pressure measurements. Identifying patients with a reverse-dipper profile may help guide more intensive therapeutic strategies aimed at slowing CKD progression and reducing cardiovascular risk. These results are exploratory and should be interpreted cautiously due to the limited sample size and observational design. Larger studies are needed to confirm these associations.

## 6. Limitations

The limitations of this study include the relatively small sample size, which might hinder the generalizability of the findings. The observation period (6 months) might also be too short to detect changes in echocardiography readings. Also, the study did not analyze the occurrence of orthostatic hypotension in patients.

## Figures and Tables

**Table 1:** Clinical characteristics of CKD patients with “dipper + extreme dipper’’, “non-dipper’’ and “reverse dipper’’ blood pressure profile at baseline and after 6 months.

Parameter	Dipper + Extreme Dipper (N= 31)	Non-Dipper (N= 41)	Reverse Dipper (N = 15)	ANOVA *p*
Women (men)	15(16)	17(24)	8(7)	NS
Age [years]	54 ±12	57 ±11	63 ±8 ^a^	0.0272
BMI [kg/m^2^]				
Baseline	26.2 ± 3.2	27.5 ±4.1	28.6± 4.3	0.11
After 6 months	26.4 ± 3.2	27.3 ±3.9	28.5 ± 4.6	0.22
Total cholesterol [mmol/L]	206.3 ± 45 219.6 ± 51.9	193.7 ± 52.0 212 ± 54.9	199.6 ±47.8 216.9 ± 41.3 *	0.56
Triglycerides [mmol/L]	135.2 ±76.4 114.5 ± 47.0	148.7 ± 63.5 147.4± 66.0	122.3 ± 46.5 134.3 ± 53.6	0.19
Creatinine [mg/dL]				
Baseline	0.98 ± 0.29	1.07 ± 0.38	1.16 ±0.39	0.24
After 6 months	0.99 ± 0.32	1.16 ± 0.53 *	1.26 ± 0.45	0.13
eGFR CKD-EPI [mL/min/1.73 m^2^]				
Baseline	80.6 ± 23.1	74.8 ± 23.6	63.4 ± 21.0	0.071
After 6 months	80.2 ± 23.8	71.4 ± 26.1	58.3 ± 21.8 ^a^	0.021
SBP 24 h [mmHg]				
Baseline	124.0 ± 11.7	126.0 ± 14.9	130.9 ± 13.3	0.28
After 6 months	123.1 ± 12.9	127.2 ± 14.1	131.5 ± 14.1	0.16
DBP 24 h [mmHg]				
Baseline	75.6 ± 8.4	75.5 ± 9.3	76.4 ± 6.7	0.94
After 6 months	75.2 ± 6.8	76.5 ± 10.0	75.8 ± 6.2	0.81
SBP day [mmHg]				
Baseline	133.3 ± 12.5	128.5 ± 14.8	129.1 ± 13.5	0.36
After 6 months	128.7 ± 13.2	128.1 ± 22.6	134.9 ± 13.3	0.46
DBP day [mmHg]				
Baseline	82.6 ± 7.5	77.8 ± 9.8 ^b^	76.9 ± 7.3 ^a^	0.025
After 6 months	78.9 ± 6.8 *	77.5 ± 13.6	78.4 ± 5.8	0.86
SBP night [mmHg]				
Baseline	112.7 ± 10.5	122.1 ± 14.9 ^b^	133.2 ± 12.9 ^a,c^	0.000022
After 6 months	112.3 ± 13.2	115.6 ± 22.3	124.3 ± 16.8 *	0.14
DBP night [mmHg]				
Baseline	67.6 ± 7.0	72.6 ± 9.9 ^b^	76.0 ± 6.7 ^a^	0.005
After 6 months	66.9 ± 7.5	68.5 ± 13.4	70.8 ± 7.5	0.52
LVMI [g/m^2^]				
Baseline	96.2 ± 24.6	108.5 ± 25.0	103.7 ± 29.3	0.14
After 6 months	93.8 ± 25.9	108.3 ± 24.6	97.8 ± 29.7 *	0.058
RWT				
Baseline	0.39 ± 0.07	0.43 ± 0.07	0.43 ± 0.07	0.10
After 6 months	0.39 ± 0.07	0.41 ± 0.06 *	0.40 ± 0.07	0.71
EF [%]				
Baseline	65 ± 7	64 ± 9	62 ± 8	0.69
After 6 months	64 ± 6	63 ± 8	58 ± 4 ^a^	0.03
IMT [mm]				
Baseline	0.63 ± 0.13	0.65 ± 0.15	0.74 ± 0.12	0.049
After 6 months	0.62 ± 0.13	0.66 ± 0.14	0.73 ± 0.12	0.043
PWV [m/s]				
Baseline	9.03 ± 1.66	9.58 ± 2.74	8.97 ± 1.59	0.49
After 6 months	8.56 ± 1.47	9.47 ± 2.43	9.52 ± 1.94	0.15

Abbreviations: BMI—body mass index; SBP—systolic blood pressure; DBP—diastolic blood pressure; EF—ejection fraction; LVMI—left ventricular mass index; RWT—relative wall thickness of the left ventricle; IMT—intima-media thickness, PWV—Pulse Wave Velocity. * Baseline vs. after 6 months *p* < 0.05; ^a^ Reverse dipper vs. dipper + extreme dipper *p* < 0.05; ^b^ Non-dipper vs. dipper + extreme dipper *p* < 0.05; ^c^ Reverse dipper vs. non-dipper *p* < 0.05.

**Table 2:** Results of multiple regression analysis for parameters of subclinical damage at baseline and after 6 months. (**a**) PWV as the dependent variable. (**b**) LVMI as the dependent variable. (**c**) EF as the dependent variable. (**d**) IMT as the dependent variable.

(a)							
Baseline R = 0.62; R^2^ = 0.38; Adjusted R^2^ = 0.34; F = 9.73; *p* = 0.0000				After 6 Months R = 0.74; R^2^ = 0.39; Adjusted R^2^ = 0.50; F = 12.4; *p* = 0.0000			
Independent variables	Beta	Beta standard error	*p*	Independent variables	Beta	Beta standard error	*p*
Age	0.47	0.09	0.000003	Age	0.55	0.11	0.000006
SBP 24 h	0.32	0.09	0.0011	SBP 24 h	0.38	0.14	0.0061
Reverse dipper	−0.22	0.09	0.0196	Gender	0.25	0.09	0.0048
Gender	0.20	0.099	0.0458	DBP 24 h	−0.19	0.13	0.1728
Total cholesterol	0.14	0.098	0.1487	Non-dipper	0.13	0.11	0.1198
				Duration of hypertension	−0.19	0.11	0.0910
				eGFR CKD-EPI	−0.12	0.10	0.24082
(**b**)							
**Baseline** **R = 0.63; R^2^ = 0.39; Adjusted R^2^ = 0.35; F = 8.56; *p* = 0.0000**				**After 6 Months** **R = 0.68; R^2^ = 0.46; Adjusted R^2^ = 0.42; F = 12.7; *p* = 0.0000**			
Independent variables	Beta	Beta standard error	*p*	Independent variables	Beta	Beta standard error	*p*
Gender	0.44	0.09	0.00001	Gender	0.35	0.10	0.1791
SBP 24 h	0.40	0.15	0.0079	SBP 24 h	0.267	0.09	0.0007
Non-dipper	−0.17	0.09	0.0618	Non-dipper	0.20	0.09	0.0210
eGFR CKD-EPI	−0.17	0.10	0.0966	Duration of hypertension	0.17	0.09	0.0792
DBP 24 h	−0.22	0.15	0.1428	Total cholesterol	−0.12	0.10	0.230
Age	−0.12	0.11	0.2931				
(**c**)							
**Baseline** **R = 0.45; R^2^ = 0.20; Adjusted R^2^ = 0.14; F = 3.36; *p* = 0.005267**				**After 6 Months** **R = 0.47; R^2^ = 0.22; Adjusted R^2^ = 0.18; F = 12.71; *p* = 0.0000**			
Independent variables	Beta	Beta standard error	*p*	Independent variables	Beta	Beta standard error	*p*
Gender	−0.31	0.11	0.00000001	eGFR CKD-EPI	0.25	0.11	0.0000000
Age	−0.30	0.14	0.0347	Gender	−0.22	0.11	0.0403
Triglycerides	−0.17	0.09	0.1022	Reverse-dipper	−0.20	0.11	0.0585
DBP 24 h	−0.18	0.11	0.1210	SBP 24 h	−0.11	0.11	0.3184
Duration of hypertension	0.27	0.14	0.0612				
eGFR CKD -EPI	0.14	0.13	0.2860				
(**d**)							
**Baseline** **R = 0.73; R^2^ = 0.39; Adjusted R^2^ = 0.54; F = 18.81; *p* = 0.0000**				**After 6 Months** **R = 0.73; R^2^ = 0.54; Adjusted R^2^ = 0.52; F = 22.54; *p* = 0.0000**			
Independent variables	Beta	Beta standard error	*p*	Independent variables	Beta	Beta standard error	*p*
Age	0.64	0.08	0.000000	Age	0.68	0.09	0.000000
SBP 24 h	0.17	0.08	0.0445	SBP 24 h	0.27	0.08	0.0010
Triglycerides	0.17	0.08	0.0296	Total cholesterol	−0.11	0.08	0.1643
Gender	0.10	0.08	0.2178	eGFR CKD-EPI	0.11	0.09	0.2505
Reverse-dipper	0.085	0.08	0.2951				

Abbreviations: PWV—Pulse Wave Velocity; SBP—systolic blood pressure; LVMI—left ventricular mass index; eGFR CKD-EPI—Glomerular Filtration Rate Estimation by CKD-EPI; DBP—diastolic blood pressure; EF—ejection fraction; IMT—intima-media thickness.

## Data Availability

The raw data supporting the conclusions of this article will be made available by the authors, without undue reservation. All data generated or analyzed during this study are included in this published article.
